# A Unique Presentation of Metastatic Gallbladder Carcinoma

**DOI:** 10.1155/2021/6455540

**Published:** 2021-12-16

**Authors:** Yuqian Tian, Carrie Luu, Danielle H. Carpenter, Grace Montenegro

**Affiliations:** ^1^Saint Louis University School of Medicine, USA; ^2^Department of Surgery, SSM Health Saint Louis University Hospital, USA; ^3^Department of Pathology, SSM Health Saint Louis University Hospital, USA

## Abstract

Gallbladder carcinoma can be challenging to diagnose and treat and usually leads to poor outcome, due to its aggressive nature and the nonspecific clinical presentation at early stage. We describe an interesting case of a 60-year-old female who presented with stage 3 appendiceal carcinoma after appendectomy was performed outside hospital. Further imaging workup demonstrated enlarged ovarian cysts and porcelain gallbladder. Upon exploration, she was found to have carcinomatosis and we proceeded with cytoreductive surgery (CRS) and hyperthermic intraperitoneal therapy (HIPEC). Final pathology demonstrated carcinoma from gallbladder primary.

## 1. Introduction

Porcelain gallbladder is a term describing calcification of the gallbladder wall. The name is given because extensive calcium deposits in the gallbladder wall have a strong resemblance to “porcelain.” [1, 2] Porcelain gallbladder is a concern because of its association with gallbladder carcinoma. However, the extent of this association is unclear. Historically, the incidence of malignancy in porcelain gallbladder was reported to be 7-60%, while more recent studies suggest a lower incidence of 0.8-6% [1, 3]. The recommended treatment for a symptomatic porcelain gallbladder is cholecystectomy to eliminate any risk of malignancy. However, for asymptomatic patients, it is debatable whether prophylactic cholecystectomy is superior to observation [1–4].

Gallbladder cancer is a rare malignancy, accounting for 1.2% of all cancers worldwide. More than 80% of patients were diagnosed at a more advanced stage because of the aggressive nature and the nonspecific clinical presentation. The most common sites for metastasis are the liver and regional lymph nodes. Management depends on the stage of disease. Surgical resection can potentially be curative for early-stage cancer. Systemic chemotherapy is the standard of care for advanced gallbladder cancer [1, 5, 6]. The treatment of peritoneal carcinomatosis with CRS and HIPEC has become a standard for some gastrointestinal tract cancers. However, for gallbladder carcinoma with carcinomatosis, CRS and HIPEC are controversial with only case studies and case series reported in the literature [7–9].

We present a unique case describing a 60-year-old female with porcelain gallbladder and presumed stage IV appendiceal adenocarcinoma who underwent cytoreductive surgery and HIPEC with carcinomatosis later confirmed to be gallbladder in origin.

## 2. Case Presentation

**Figure 1:**
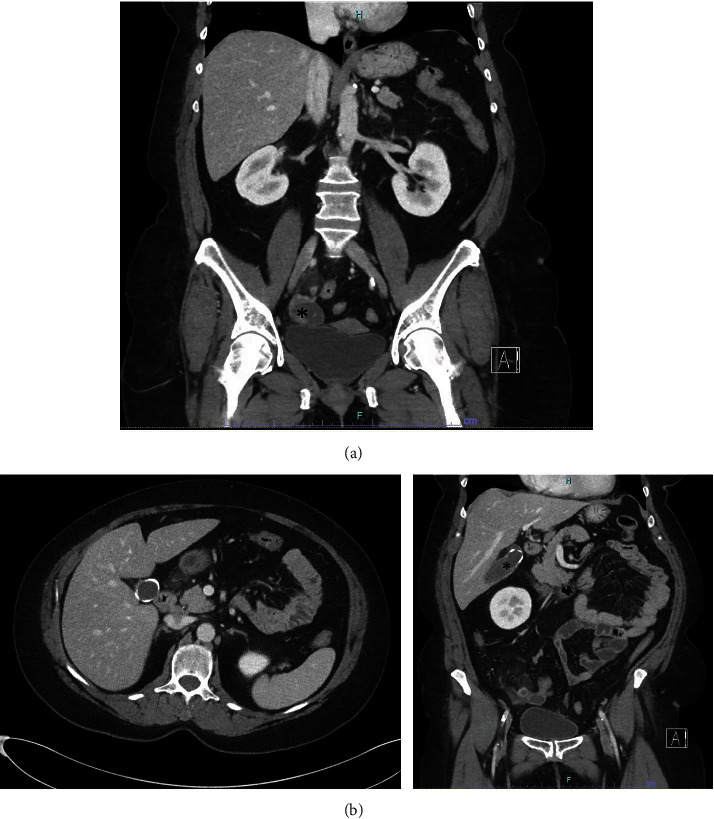
(a) CT A/P. The asterisk marks the RIGHT ovarian cyst, which was evaluated during the index operation. (b) CT A/P at OSH. The coronal view (right) and axial view (left) of porcelain gallbladder. The asterisk marks the gallbladder with a calcified wall.

**Figure 2:**
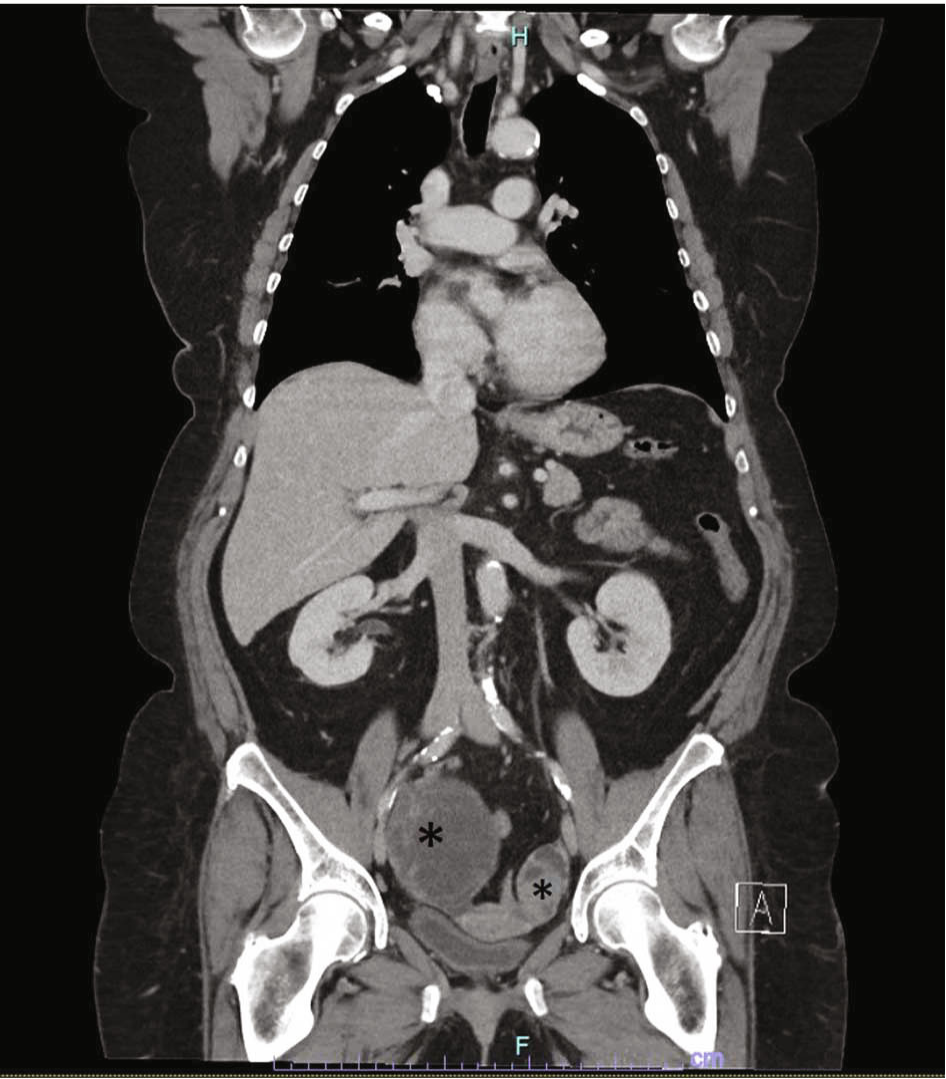
CT A/P. The asterisks mark the enlarging right ovarian lesion and a new left ovarian lesion.

A 60-year-old female presented to our tertiary medical center for a second opinion regarding the incidental pathology finding of stage III nonmucinous appendiceal adenocarcinoma after an emergent appendectomy for perforated appendicitis at an outside hospital four months prior. Her initial pathology revealed primary nonmucinous, moderately differentiated, stage III, pT4pN1aM0, appendiceal adenocarcinoma, involving 1 of 3 periappendiceal lymph nodes with extensive lymphovascular space invasion. Mismatch repair protein was intact. She completed staging computed tomography (CT) and colonoscopy. On imaging, there was no evidence of distant metastasis, but a small right ovarian cyst and calcification of the gallbladder wall were noted (Figure 1). The ovarian cyst had been evaluated intraoperatively at the index operation by a gynecologist, and it was deemed that no intervention was needed at that time. Completion right hemicolectomy and possible right oophorectomy followed by adjuvant FOLFOX (folinic acid, fluorouracil, and oxaliplatin) were recommended. However, she opted to forgo any treatment at that time. The patient was asymptomatic in the interim. The patient represented to clinic with CT findings of growth in the right ovarian cyst, from 4 to 11 cm, with a new 6 cm complex cystic/solid mass along the left pelvic sidewall (Figure 2). On presentation, she complained of lower abdominal fullness and cramping with intermittent bloating and early satiety. Her exam was mostly unremarkable except for the fullness in bilateral adnexa.

**Figure 3:**
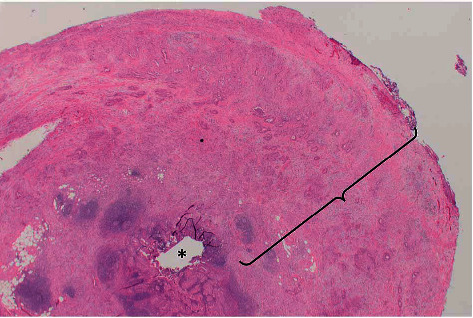
The appendiceal lumen (∗) is focally involved by adenocarcinoma, though there is an abundance of deep infiltrating carcinoma, extending onto the serosa (bracket).

Her case was presented at the multidisciplinary tumor board. At that time, her pathology was also reviewed (Figure 3). We recommended completion right hemicolectomy as well as resection of adnexal masses, which were concerning for malignancy. We also discussed the possibility of cytoreductive surgery and hyperthermic intraperitoneal chemotherapy if peritoneal metastasis was discovered on exploration. In addition, she was recommended to undergo cholecystectomy at the same time.

Intraoperatively, the patient was found to have diffuse carcinomatosis. Cytoreductive surgery included right hemicolectomy, cholecystectomy, and total abdominal hysterectomy and bilateral salpingo-oophorectomy with en bloc resection of the adnexal masses. This was followed by HIPEC with mitomycin C. The peritoneal carcinomatosis index (PCI) was 20, and the completeness of cytoreduction score (CC) was 1 due to subcentimeter implants on the small bowel serosa from the jejunum to the terminal ileum. The patient had an uneventful postoperative recovery and was discharged on postoperative day 6.

**Figure 4:**
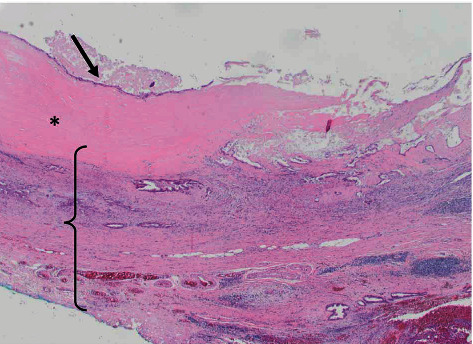
Sections of the gallbladder showed thickened hyalinized wall (∗), infiltrated by invasive carcinoma (bracket), and associated widespread high grade biliary intraepithelial neoplasia (arrow).

Surprisingly, pathology revealed primary gallbladder adenocarcinoma, moderately to poorly differentiated, arising in a background of high grade biliary intraepithelial neoplasm and porcelain gallbladder. The carcinoma extended through the visceral peritoneum onto the serosal surface and into the pericystic soft tissue on the hepatic bed surface (Figure 4). All tumor deposits collected from the operation were consistent with metastasis from the biliary origin.

**Figure 5:**
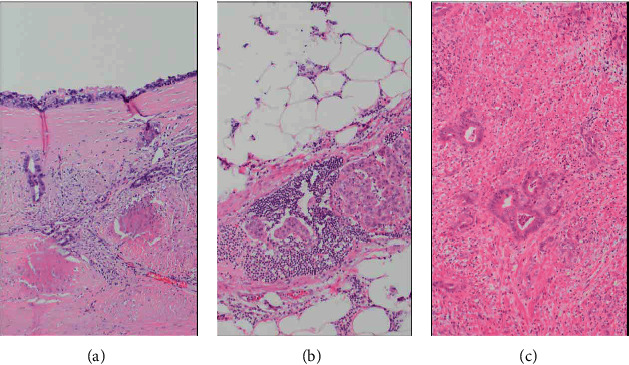
The gallbladder carcinoma (a) infiltrating the fibrotic wall of the gallbladder, with overlying high grade biliary intraepithelial neoplasia, has the same morphologic appearance as the appendiceal carcinoma (c), which had an abundance of lymphovascular space invasion (b).

With this new finding, her case was rediscussed at tumor board. The original appendiceal specimen slides were reviewed and found to be similar histologically to the gallbladder adenocarcinoma (Figure 5). The possibility of synchronous gallbladder and appendiceal primaries was discussed but given the morphological resemblance between the two and the pattern of spread, primary gallbladder adenocarcinoma with carcinomatosis was the most likely diagnosis.

The patient completed four cycles of gemcitabine and cisplatin before switching to FOLFOX after surveillance imaging demonstrated disease progression. Shortly after receiving the first cycle of FOLFOX, she presented with an acute abdomen secondary to perforated viscus and underwent emergent laparotomy. She was subsequently transitioned to hospice.

## 3. Discussion

Primary appendiceal neoplasms are incidentally found in 1-2% of appendectomies, with mucinous neoplasm (0.6%) and carcinoids (0.3-0.9) being the most common lesions [10, 11]. Metastatic involvement of the appendix presenting as acute appendicitis is an even rarer entity, with few case reports documenting such incidences from carcinoma of breast, ovary, prostate, lung, kidney, stomach, colon, and hepatobiliary tract [12–18]. Unlike the growth pattern typical of primary appendiceal carcinoma, which starts from the mucosa and progresses outward, metastasis to the appendix involves the serosa first, followed by inward infiltration [14, 16].

There are only two case reports found in the literature documenting gallbladder cancer metastasis to the appendix and only one of them presented as acute appendicitis clinically [16, 18]. Another case report described a metastatic cholangiocarcinoma mimicking acute appendicitis [17]. Our patient presented with porcelain gallbladder without any gallbladder mass or liver involvement. Therefore, the concern for gallbladder malignancy was low. In addition, she already had an appendectomy with a report of adenocarcinoma from appendiceal primary. As such, management was focused on treating the appendiceal adenocarcinoma and potential ovarian and peritoneal spread. Removal of the gallbladder was recommended at the same time due to the uncertain malignant potential of calcification of the gallbladder wall.

The change of diagnosis from appendiceal primary to gallbladder primary in our case makes the selected surgical management worth discussing. For early-stage primary appendiceal adenocarcinoma, the widely accepted surgical intervention is a right hemicolectomy, which shows a clear survival benefit when comparing to a simple appendectomy [19]. CRS and HIPEC have demonstrated improved outcomes for selected patients with peritoneal spread. However, several retrospective studies have reported mixed results regarding whether CRS and HIPEC are beneficial for nonmucinous appendiceal carcinoma [20–22]. Conversely, CRS and HIPEC are not recommended for advanced gallbladder carcinoma with peritoneal spread due to the aggressive nature of this disease. In recent years, few retrospective studies have attempted to clarify the role of CRS and HIPEC in gallbladder carcinoma. These studies are limited by the extremely small sample size and the retrospective, and therefore, no definitive conclusion can be drawn regarding the potential benefit of CRS and HIPEC. Randle et al. retrospectively evaluated five patients with advanced gallbladder cancer who underwent CRS and HIPEC and concluded that the procedure is feasible and can be performed safely [9]. Another retrospective multicenter study evaluated patients with peritoneal metastasis from biliary carcinoma and compared survival outcomes of CRS and HIPEC with palliative chemotherapy. They suggested that CRS and HIPEC may offer survival benefit comparing to palliative chemotherapy in highly selected patients [23].

This case presents some unique diagnostic and treatment challenges. The patient presented with a diagnosis of appendiceal carcinoma, and treatment was based on this diagnosis. Without clear imaging findings of gallbladder malignancy and lack of symptoms, the diagnosis of gallbladder carcinoma with metastasis to the appendix would not likely have been made preoperatively. In addition, the intraoperative frozen section of the gallbladder was not performed which might have spared the patient an extensive cytoreductive surgery with HIPEC. Lastly, though there are case reports demonstrating the feasibility of cytoreductive surgery and HIPEC in gallbladder carcinoma with peritoneal metastasis, as this study also does, we do not believe it should be performed for gallbladder cancer due to the aggressiveness of this disease.
